# Anti-**α**-enolase Antibodies in Serum from Pediatric Patients Affected by Inflammatory Diseases: Diagnostic and Pathogenetic Insights

**DOI:** 10.1155/2011/870214

**Published:** 2011-10-05

**Authors:** Alessandra Pontillo, Nicola Di Toro, Paolo Edomi, A. Shadlow, A. Ammadeo, M. Gattorno, T. Not, L. Lepore, S. Crovella

**Affiliations:** ^1^Institute for Maternal and Child Health, IRCCS Burlo Garofolo, via dell'Istria 65/1, 34137 Trieste, Italy; ^2^Department of Life Science, University of Trieste, Italy; ^3^Institute for Maternal and Child Health, IRCCS Burlo Garofolo, and University of Trieste, via dell'Istria 65/1, 34137 Trieste, Italy; ^4^Pediatric Unit II, Institute of Child Health, IRCCS G. Gaslini, Genoa, Italy

## Abstract

Human glycolytic enzyme *α*-enolase was associated with human diseases and with inflammation. An ELISA test was developed to measure anti-*α*-enolase AAE IgG and AAE IgA in the serum from patients affected by inflammatory diseases with the purpose to evaluate it as a novel diagnostic marker. 80 healthy blood donors and 194 paediatric patients affected by Juvenile idiopathic arthritis (JIA), celiac disease (CD), Crohn's Disease (CrD), hereditary periodic fever (HPF), and PFAPA syndrome were included in the study. HPF patients showed high levels of AAE antibodies, whereas JIA, CD, and CrD presented only partial results. Benign fevers such as PFAPA were almost negative for AAE Abs. These findings suggested that the genetic dysfunction of inflammasome associated with HPF could lead to the formation of AAE Abs that could be used for an early and easy diagnosis.

## 1. Introduction

The human glycolytic enzyme *α*-Enolase was previously associated with human diseases (i.e., Crohn's disease and Systemic Lupus Erythematosus), as a novel autoantigen with an unknown pathologic significance other than tissue damage, or cross-reactivity after a contact with bacterial or yeast enolase [1–6]. 

*α*-Enolase undergoes a posttranslational modification known as arginine deimination or citrullination by peptidyl-arginine-deiminase (PAD). Anticitrullinated *α*-Enolase antibodies were found in serum of different immune diseases, that is, rheumatoid arthritis (RA), multiple sclerosis, Alzheimer's disease, and psoriasis [7–9]. 

Recently it was shown that during the inflammatory process, *α*-Enolase, together with other glycolytic enzymes, might be a substrate of caspase-1 in the newly described caspase-1 digestosome [10]. Caspase-1 leads to the activation of IL-1*β* and NF-kB, through the inflammasome, and also to the inflammatory cell death called pyroptosis. Pyroptosis, eliminating macrophages, probably contributes to the end of inflammation. Because glycolysis is essential for macrophage survival and activation, the cleavage of *α*-Enolase and the glycolysis substrates, which results in reduction of cellular glycolysis, seems to be an essential step toward cell death [10].

We hypothesized that high amount of *α*-Enolase-derived peptides and/or their inefficient removal from the milieu could be immunogenic and that high amount of antibodies anti-*α*-enolase could be an epiphenomenon in inflammatory processes that are characterized by an important caspase-1 activation. The evaluation of antibodies anti-*α*-enolase (AAE Abs) could provide a novel tool for better definition of differential diagnosis in patients with inflammatory disorders.

For this purpose we set up an Enzyme-Linked Immune Sorbent Assay (ELISA) to evaluate serum anti-*α*-enolase antibodies (AAE Abs) in pediatric healthy controls and patients affected by chronic inflammatory conditions due to different ethiopathogenesis: Juvenile idiopathic arthritis (JIA), celiac disease (CD), Crohn's disease (CrD), and periodic fevers, namely chronic infantile neurologic cutaneous and articular (CINCA) syndrome, mevalonate kinase deficiency (MKD), Familial Mediterranean fever (FMF), TNF-R associated periodic syndrome (TRAPS), and periodic fever associated to aphthous, pharyngitis, and cervical adenopathies (PFAPA).

## 2. Materials and Methods

### 2.1. Patients

194 sera from children and adolescents with a diagnosis of inflammatory disease were included in the study. 31 patients are affected with Juvenile Rheumatoid Arthritis (11 males/20 female; 11.2 years ± 4 standard deviation, SD), 55 with celiac disease (17 males/38 females; 12.7 ± 11.3), 59 with Crohn's disease (33 male/26 female; 11.9 ± 5.1), 20 with hereditary periodic fever (HPF; 13 male/7 female; 12.6 ± 8.1), and 28 with PFAPA syndrome (PFAPA; 13 male/15 female; 3.4 ± 2.7). The group of HPF consists of 9 CINCA syndrome, 5 FMF, 5 MKD and 1 TRAPS.

184 patients were recruited from the Paediatric Division of the Institute of Maternal and Child Health IRCCS “Burlo Garofolo” (Trieste, Italy) and 10 (5 FMF and 5 CINCA) from the II Paediatric Division of the Institute of Child Health IRCCS “G. Gaslini” (Genoa, Italy). All the sera were collected at the moment of the diagnosis, before starting any kind of treatment. 

JIA was diagnosed according to the criteria of the International League of Associations for Rheumatology [11]. The CD diagnosis was based on the evaluation of clinical features, ELISA anti-transglutaminase antibody (tTG) quantification (Eu-tTG kit, Eurospital, Trieste, Italy), and the presence of the HLA DQ2/DQ8 heterodimer (Eu-Gen Risk, Eurospital) and confirmed by intestinal biopsy following the indications recommended by the European Society for Paediatric Gastroenterology, Hepatology and Nutrition [12]. CrD was diagnosed according to clinical, endoscopic, and histologic criteria, as suggested by international guidelines [13]. HPFs were diagnosed according to clinical parameters specifically related to each kind of disease [14] and to the molecular analysis of respective genes (CINCA syndrome: *CIAS1/NALP3; *FMF:* MEFV; *MKD* MVK; *TRAPS: *TNFRS1A*). PFAPA syndrome was diagnosed by the meaning of clinical signs [15] and after the exclusion of any HPF by the meaning of the analysis of the HPF genes.

Viral or pathogen infections as well as cancer have been previously excluded. 

Moreover, 80 healthy Italian subjects (35 males/45 females; mean age 7.1 years ± 4.8 SD) matched for age and ethnicity with the patients, and not related to the patients group, were included as controls (HC). One patient affected by vasculitis was also included as positive control [16].

The study protocol was approved by independent ethics committees at each study site. Patients or their parents provided written informed consent.

### 2.2. ELISA for Anti-*α*-enolase IgG and IgA Antibodies

Two ELISA tests were developed using recombinant *α*-Enolase (a gift from Dr. Edomi; University of Trieste) as the capture reagent to detect AAE IgA or AAE IgG serum antibodies. Phosphatase-conjugated anti-human IgA or anti-human IgG (*Sigma Aldrich*) were used as the detection antibody.

Microtiter plates (96-well *Nunc Maxisorp*) were coated with purified unlabelled recombinant *α*-Enolase at 9.5 *μ*g/mL in PBS overnight at 4°C. Plates were then washed with 0.05% Tween-20 in PBS (wash buffer) and saturated with 0.1% Tween-20 in PBS for 20°C at room temperature (RT). 

The ELISA was performed by diluting healthy controls and patients serum 1 : 100 in PBS for IgG test and 1 : 200 for IgA test. The serum from children affected by vasculitis was used as positive control as previously reported [16]. All incubations were performed at RT, and all volumes were 100 *μ*l per well. The plates were incubated for 1 hour and washed three times with wash buffer. 

The phosphatise-conjugated anti-human IgA (diluted 1 : 2000; *Sigma Aldrich*) or anti-human IgG (diluted 1 : 2000; *Sigma Aldrich*) was then added to the wells, incubated for 1 hour, and plates were washed three times. The assay was developed by the addition of 1 g/L *p*-nitrophenyl phosphate (*Sigma Aldrich*) to the plate. The data was acquired by measuring absorbance at 405 nm and analysed using GraphPad Prism version 5.0. Results were expressed as arbitrary unit (AU)/mL.

### 2.3. Data Analysis

Statistical analysis was carried out using the GraphPad Prism software version 5.0. For the comparison of groups, independent Student *t*-test and ANOVA with Scheffe's post hoc test were used. *P* values of less than 0.05 were regarded as significant. 

## 3. Results

Demographic characteristics (mean age, male/female ratio) were compared between healthy controls (HCs) and each group of patients and data were reported in Table 1.

80 sera from healthy blood donors included as controls were tested for the presence of AAE IgA and IgG antibodies. The mean and standard deviation (SD) was 15.44 UA/mL ± 5.21 for AAE IgA and 16.79 UA/mL ± 5.81 for AAE IgG.

The cut-off values calculated for each Ig class (mean + 2SD) were 25.86 and 28.41, respectively.

The results for the vasculitis patient serum were 29.92 AU/mL for AAE IgA and 54.08 AU/mL for AAE IgG.

A larger number of serum samples from patients with chronic inflammatory diseases were procured to test the utility of the ELISA in detecting AAE antibodies.

Samples of sera from 194 paediatric patients affected by JIA (*n* = 31), CD (*n* = 55), CrD (*n* = 59), HPF (*n* = 20), and PFAPA (*n* = 29) were compared with the controls sera.

 The mean and standard deviation of each group for the assays were as follows: JIA IgA: 31.21 ± 22.11; IgG: 42.52 ± 13.28, for CD 25.72 ± 24.74; 34.23 ± 12.42, CrD 29.60 ± 16.69; 43.04 ± 18.39, for HPF 37.41 ± 34.51; 46.57 ± 21.21, for PFAPA 11.59 ± 7.87; 14.09 ± 6.46 (Table 1). Using the unpaired *t*-test (2-tailed) method to evaluate the difference between the HC and patient sera groups, the differences as follow: each comparison were HC versus JIA IgA: *P* = 4.4exp−4; IgG: *P* = 3.43exp−12, for HC versus CD *P* = 0.004; *P* = 1.28exp−14, for HC versus CrD *P* = 2.81exp−8; *P* = 6.7exp−16, for HC versus HPF *P* = 0.01; *P* = 8.43exp−6, for HC versus PFAPA *P* = 0.02; *P* = 0.05 (Table 2). 

Among the patient groups significant differences were reported for PFAPA versus all the other groups as AAE IgA and IgG, JIA versus CD (IgG: *P* = 0.006), CD versus CrD (IgG: *P* = 0.003), and CrD versus HPF (IgG: *P* = 0.025) (See Supplementary Data S1 in supplementary material available online at doi: 10.1155/2011/870214). 

**Figure 1:**
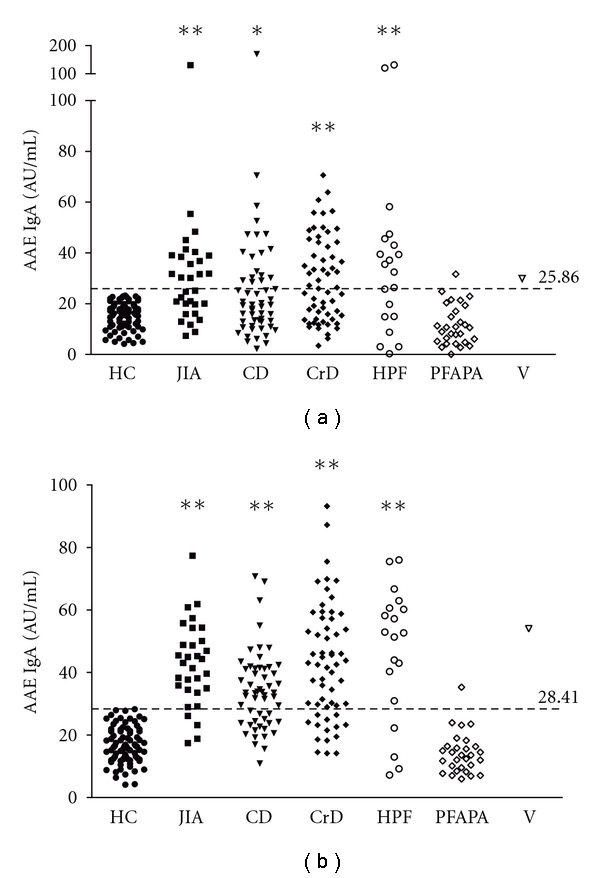
Levels of anti-*α*-enolase antibodies ((a) :  IgG; (b) : IgA) in patients with juvenile idiopathic arthritis (JIA), celiac disease (CD), Crohn's disease (CrD), hereditary periodic fevers (HPF), PFAPA syndrome (PFAPA), and vasculitis (V) and healthy controls (HC). Results are expressed as arbitrary units per mL (AU/mL). Cut-off values are indicated with a dashed line. Patients' antibody levels are compared to healthy control by *t*-test (∗∗ = *P *< 0,001, ∗ = *P *< 0,05).

Furthermore, using the cut-off values of 25.86 AU/mL for IgA, 52% (16 of 31 samples) of JIA, 33% (18/55) of CD, 53% (31/59) of CrD, 65% (13/20) of HPF, and 4% (1/29) of PFAPA sera were above the cut-off (AAE IgA+) (Figure 1(a), Table 2). 

87% (27/31) of JIA, 65% (36/55) of CD, 75% (44/59) of CrD, 80% (16/20) of HPF, and 4% (1/29) PFAPA sera were above the IgG cut-off of 28.41 AU/mL (AAE IgG+) (Figure 1(b), Table 2). 16 out of 31 JIA (52%), 15 out of 55 CD (27%), 24 out of 59 CD (41%), and 1 out of 20 HPF (5%) were both AAE IgA+ and AAE IgG+ (Table 2). 

Overall, the samples represent an average of 41% positive AAE IgA and 64% AAE IgG above the cut-offs established for the assay. Despite the high specificity of the AAE Abs test (100% for IgA and IgG), the sensitivity varied within the cohorts ranging from 33 to 65% for AAE IgA and from 65 to 87% for AAE IgG (Table 2). These results show that the AAE ELISA can distinguish differences between serum from healthy donors and patients with an important inflammatory disease.

When patients affected by JIA were stratified for joint involvement and severity of the disease (polyarticular, pauciarticular, and systemic JIA) a significant difference was observed for AAE IgA titre in polyarticular JIA versus HC (*P* = 2.0exp−4) and poliarticular JIA versus systemic JIA (*P* = 0.004); for AAE IgG all the subgroups were significantly different compared to HC (Table 3).

13 out of 16 patients with pauci-articular JIA were AAE IgG+ (81%) and 7 were AAE IgG+ IgA+ (44%). 9 out of 10 subjects with a poliarticular JIA were AAE IgG+ (90%) and 8 AAE IgG+ IgA+ (80%). The 5 patients with systemic arthritis were AAE IgG+ (100%), and only 1 out of 5 was AAE IgG+IgA+ (20%) (Table 3). The AAE evaluation did not vary according to the clinical manifestation of JIA, with the only exception of AAE IgA level that is significantly higher in polyarticular compared to systemic JIA (*P* = 0.035) (Table 3).

Among the group of hereditary periodic fever, 67% of CINCA patients (6/9) were AAE IgA+ and all the 9 CINCA patients (100%) were AAE IgG+. 80% (4/5) of FMF were both AAE IgA+ IgG+, and 20% (1/5) were double negative for the test. 60% (3/5) of MKD were AAE IgA+, 60% (3/5) were AAE IgG+, and 40% (2/5) were AAE IgA+ IgG+. The results of 1 MKD patient, to note the one with the most severe clinical presentation, were fully negative. The TRAPS patient serum resulted in being negative for the AAE detection. Also in this cohort the ELISA results did not report any significant difference within the 4 HPFs (Table 3). 

## 4. Discussion

This study showed that autoantibodies directed against human *α*-Enolase are present in different inflammatory conditions in paediatric patients, such as juvenile idiopathic arthritis, celiac disease, Crohn's disease, and hereditary periodic fevers, but not in benign fever PFAPA. Initially described as diagnostic marker for rheumatoid arthritis (RA), AAE antibodies seem to occur in several inflammatory disorders [17], lacking the proposed role of specific diagnostic test for RA. 

As previously reported for RA in adults, AAE antibodies were present in JIA. In particular, AAE IgG was found in 87% of JIA sera (Table 2) in respect to 25% and 6% of positivity in RA sera demonstrated with immune-blotting technique by other authors [18, 19]. The rate of positive sera among our paediatric group was particularly high in the systemic presentation of the disease (AAE IgG 100%), whereas AAE IgA levels were more heterogeneous (Table 3). Although JIA does not present mucosa involvement, these results could be explicated taking in account a hypothesis of polyclonal activation in this inflammatory disease with autoimmune etiopathology, as described by other authors [17]. Moreover the presence of AAE Abs did not correlate with a bad prognosis for patients with JIA as suggested by Saulot in early rheumatoid arthritis [19], even if we could only observe patients in a short follow-up period (6–12 months).

AAE antibodies were previously described, with a proteomic approach, in adults affected by celiac disease [5] and by inflammatory bowel disease [20]. In celiac subjects we observed a low titre of AAE IgA (33%) and a quite elevated one of AAE IgG (65%) (Table 2). This suggests that the activation of autoreactive B cells against *α*-Enolase could be not mucosa specific, but perhaps a systemic event. 

74% of Crohn's patients presented AAE IgG compared to data reported by Vermeulen et al. (AAE IgG 50%) [20], highlighting the massive inflammatory state of these subjects.

These results confirm previously reported findings about the presence of AAE Abs in the serum of patients affected by important inflammatory conditions, although the variable sensitivity of the test does not sustain our initial enthusiasm.

For the first time to our knowledge AAE antibodies were described in subjects affected by recurrent fevers. Our results clearly showed a difference between benign fever PFAPA, which was almost negative for AAE Abs, and hereditary periodic fevers (Table 2). Moreover, among the HPFs, the ones characterized by a genetic defect in inflammasome—such as CINCA syndrome and Familial Mediterranean fever—are more frequently positive for the test (Table 3). 

Hereditary periodic fevers represent a big diagnostic challenge due to the wide and often unspecific clinical spectrum that accounts for possible difficulties in differential diagnosis with chronic inflammatory disorders and also with the benign PFAPA syndrome. However an early diagnosis is very important in these conditions to start the appropriate treatment and to prevent unwanted long-term complications in HPF or, on the other hand, to rapidly exclude a severe disease in PFAPA and avoid inappropriate and often expensive investigations. For this reason, despite the limited size of our cohorts, we believe that AAE antibodies titre could be a novel useful inflammatory marker for these rare syndromes. 

Finally, our findings are interesting also considering their ethiopathologic implications. The quite different levels of anti-*α*-enolase antibodies that we found in our patients seem to support our hypothesis that the immunogenicity of *α*-Enolase could be a characteristic event in caspase-1-related diseases. We underlined that the highest AAE Abs levels were observed in CINCA syndrome, Familial Mediterranean fever, and the inflammasome-related syndromes. HIDS patients presented high AAE Abs, and this disease was recently associated with the autoactivation of caspase-1, too [21]. 

Moreover, one of the most known susceptibility genes for Crohn's disease is *NOD2*. *NOD2* belongs to the NOD-Like Receptor (NLR) superfamily comprising also *CIAS1/NALP3* (mutated in CINCA syndrome). *NOD2* induces NF-kB, similarly to inflammasome, and it was supposed to activate caspase-1 interacting with NALP1 [22], so it is possible that the 2 signalling cascades (inflammasome and NOD-some), could converge and affect the same molecular targets, such as glycolytic enzymes destruction before cell death. A dysfunction in inflammasome was also hypothesized for early rheumatoid arthritis [23] and celiac disease [24] but with discordant results. For PFAPA syndrome several studies were made to elucidate the genetic association with known inflammatory genes, but without any findings. Considering its benign clinical presentation, its good prognosis, and the lack of association with any known inflammatory gene, nowadays PFAPA syndrome is not included in hereditary autoinflammatory disorders. We hypothesized that in these patients the inflammasome pathway and caspase-1 are still untouched and for this reason the pathologic events that lead to the high production of AAE Abs do not occur.

In conclusion, paediatric patients affected by hereditary periodic fevers showed high levels of AAE Abs; JIA, Crohn's disease, and celiac disease presented only partial results, whereas benign fevers such as PFAPA were almost negative.

Although our results are quite preliminary and obtained with a yet unvalidated technique and need further investigation as well as analyses performed on more numerically consistent cohorts of healthy controls and patients, we believe that AAE Abs evaluation could represent a quite cheap and fast way to characterize subjects with an important inflammatory disregulation, especially in a differential diagnosis between hereditary periodic fevers and benign fever such as PFAPA syndrome. For these auto-inflammatory syndromes the only specific laboratory investigation is the mutational screening in candidate genes, which is time-spending and expensive, especially considering that, in particular in the first years of life, recurrent fevers—variably associated with abdominal involvement and lymphadenopathy—are often present in children and they do not ever represent a genetic disease.

## Supplementary Material

Supplementary Table: T-test results from comparison within patients groups.

## Figures and Tables

**Table 1:** Patients and controls demographic data. Mean age and males/female ratio were reported for healthy controls (HC) and in patients with juvenile idiopathic arthritis (JIA), celiac disease (CD), Crohn's disease (ChD), hereditary periodic fevers (HPFs), and PFAPA syndrome (PFAPA). Age and males/females ratio from patients were compared with HC using an independent *t*-test and a Fisher exact test, respectively.

	Age (years; mean ± SD)	*P*	Males/females ratio	*P*
HC (*n* = 80)	7.1 ± 4.8		35/45	
JIA (*n* = 31)	11.2 ± 4	0.12	11/20	0.52
CD (*n* = 55)	12.7 ± 11.3	0.31	17/38	0.15
CrD (*n* = 59)	11.9 ± 5.1	0.10	33/26	0.17
HPF (*n* = 20)	12.6 ± 8.1	0.14	13/7	0.13
PFAPA (*n* = 29)	3.4 ± 2.7	0.08	13/15	0.83

**Table 2:** AAE IgA and AAE IgG in healthy controls and patients. Levels and percentage of positivity of anti-*α*-enolase IgA and IgG in healthy controls (HCs) and patients with juvenile idiopathic arthritis (JIA), celiac disease (CD), Crohn's disease (ChD), hereditary periodic fevers (HPF), and PFAPA syndrome (PFAPA) were reported as mean and standard deviation (SD). AAE levels are expressed as arbitrary units per mL (AU/mL) and AAE positivity as number and percentage of sera with titre above the cut-off. Patients' AAE levels are compared to healthy control by *t*-test and *P* values are reported.

	AAE IgA	AAE IgA+	*P*	AAE IgG	AAE IgG+	*P*
HC (*n* = 80)	15.44 ± 5.21	0		16.79 ± 5.81	0	
JIA (*n* = 31)	31.21 ± 22.11	16 (52%)	4.4exp−4	42.52 ± 13.28	27 (87%)	3.4exp−12
CD (*n* = 55)	25.72 ± 24.74	18 (33%)	0.004	34.23 ± 12.42	36 (65%)	1.3exp−14
CrD (*n* = 59)	29.60 ± 16.69	31 (53%)	2.8exp−8	43.04 ± 18.39	44 (75%)	6.7exp−16
HPF (*n* = 20)	37.41 ± 34.51	13 (65%)	0.01	46.57 ± 21.21	16 (80%)	8.4exp−6
PFAPA (*n* = 29)	11.59 ± 7.87	1 (4%)	0.02	14.09 ± 6.46	1 (4%)	0.05

**Table 3:** Serum level and percentage of AAE IgA+ and AAE IgG+ in JIA stratified for clinical presentation and in HPF classified for genetic defect. Levels of anti-*α*-enolase IgA and IgG in patients with pauciarticular, polyarticular, or systemic juvenile idiopathic arthritis (JIA) CINCA syndrome, FMF, MKD, and TRAPS hereditary periodic fevers (HPF) were reported as mean and standard deviation (SD). Results are expressed as arbitrary units per mL (AU/mL). Patients' AAE levels are compared by *t*-test (∗ = *P *< 0.05). Percentage of AAE IgA and IgG positivity in each disease class is also reported.

	AAE IgA (mean + SD)	AAE IgG (mean + SD)	IgA+	IgG+	IgA+ IgG+
JIA					
JIA-pauci (*n* = 16)	30.09 ± 28.77	39.75 ± 13.20	7 (44%)	13 (81%)	7 (44%)
JIA-poly (*n* = 10)	36.98 ± 11.62*	49.09 ± 14.59	8 (80%)	9 (90%)	8 (80%)
JIA-syst (*n* = 5)	23.26 ± 10.0*	38.23 ± 5.38	1 (20%)	5 (100%)	1 (20%)
HPF					
CINCA (*n* = 9)	29.58 ± 16.47	55.06 ± 12.81	6 (67%)	9 (100%)	6 (67%)
FMF (*n* = 5)	49.30 ± 43.43	48.43 ± 29.16	4 (80%)	4 (80%)	4 (80%)
MKD (*n* = 5)	45.33 ± 51.28	36.52 ± 21.99	3 (60%)	3 (60%)	2 (40%)
TRAPS (*n* = 1)	8.77	12.97	—	—	—
