# Supplementary material for: Anti-α-enolase Antibodies in Serum from Pediatric Patients Affected by Inflammatory Diseases: Diagnostic and Pathogenetic Insights
Source: Int J Rheumatol. 2011 Oct 5;2011:870214. doi: 10.1155/2011/870214 (PMC3189593; doi:10.1155/2011/870214)
Supplement: Supplementary file 1 — Supplementary Table: T-test results from comparison within patients groups. [file 870214.f1.pdf]

Supplementary data S1. T-test results from comparison within patients groups. Patients AAE levels are compared within the different cohorts by t-test and p value were reported. In the right side of the table were reported results for AAE IgG, whereas in the left side the ones for AAE IgA. In bold are indicated $p<0.05$.

IgG / IgA	JIA	CD	CrD	HPF	PFAPA
JIA		**0.006**	0.879	0.461	**9.1 exp-14**
CD	0.294		**0.003**	**0.025**	**2.1 exp-15**
CrD	0.724	0.332		0.520	**2.5 exp-17**
HPF	0.481	0.176	0.341		**2.4 exp-6**
PFAPA	**4.1 exp-5**	**2.0 exp-4**	**9.2 exp-10**	**0.004**	
